# Change in Medication Adherence and Beliefs in Medicines Over Time in Older Adults

**DOI:** 10.5539/gjhs.v8n5p39

**Published:** 2015-08-31

**Authors:** Elizabeth J Unni, Olayinka O Shiyanbola, Karen B Farris

**Affiliations:** 1 College of Pharmacy, Roseman University of Health Sciences, Utah, USA; 2 School of Pharmacy, University of Wisconsin, Madison, Wisconsin, USA; 3 College of Pharmacy, University of Michigan, Ann Arbor, Michigan, USA

**Keywords:** temporal relationship, older adults, medication adherence, beliefs in medicines, counseling, early intervention

## Abstract

**Objective:**

The temporal component of medication adherence is important while designing interventions to improve medication adherence. Thus, the objective of this study was to determine how medication adherence and beliefs in medicines change over time in older adults.

**Methods:**

A two-year longitudinal internet-based survey among adults 65+ years was used to collect data on medication adherence (Morisky 4-item scale) and beliefs in medicines (Beliefs about Medicines Questionnaire). Paired t-test and one-way ANOVA determined if a change in beliefs in medicines and medication adherence over time was significant. A multiple linear regression was used to determine the significant predictors of change in medication adherence over time.

**Results:**

436 respondents answered both baseline and follow-up surveys. Among all respondents, there was no significant change in adherence (0.58 ± 0.84 vs. 0.59 ± 0.84; p > 0.05), necessity beliefs (17.13 ± 4.31 vs. 17.10 ± 4.29; p > 0.05), or concern beliefs (11.70 ± 3.73 vs. 11.68 ± 3.77; p > 0.05) over time. For older adults with lower baseline adherence, there was a statistically significant improvement in adherence (1.45 ± 0.70 vs. 0.99 ± 0.97; p < 0.05); but no change in beliefs in medicines over time. The significant predictors of change in medication adherence over time were baseline adherence and baseline concern beliefs in medicines.

**Conclusion:**

With baseline adherence and baseline concern beliefs in medicines playing a significant role in determining change in adherence behavior over time, especially in individuals with lower adherence, it is important to alleviate medication concerns at the beginning of therapy for better adherence.

## 1. Introduction

Medication adherence is an essential component of chronic disease management. Older adults often deal with multiple illnesses and multiple medicines, making the management of diseases more complicated and challenging (Gu Q, 2010; Ward & Schiller, 2013). The rate of medication adherence among older adults with chronic medications varies from 23 to 86% showing this is an important issue that needs to be considered (Schlenk, Dunbar-Jacob, & Engberg, 2004). With significant changes that happen as age advances such as physiologic changes in kidney function, leaving the work force, having caregivers in their life, memory loss or lack of physical dexterity, it is important to understand whether older adults’ adherence behavior also change over time. Among all the factors that contribute to medication adherence in older adults, patient beliefs in medicines are quite significant and shown to be paramount in improving adherence to prescribed medications (Horne & Weinman, 1999; Sirey, Greenfield, Weinberger, & Bruce, 2013; Unni & Farris, 2011; Unni, Shiyanbola, & Farris, 2013). Whether an individual is currently taking medicines or not, they often develop attitudes and beliefs about medicines. Patient’s beliefs in medicines often include beliefs about the necessity of the prescribed medication in maintaining their health and concerns about the negative effects of the prescribed medicine (Horne, 1997). Though adherence literature has established the relationship between beliefs in medicines and medication adherence, the relationship between the change in beliefs in medicines over time and adherence over time is not well established.

Several theoretical models including the health belief model have been used to explain medication adherence (Sirur, Richardson, Wishart, & Hanna, 2009). Of all the theoretical models, the one that explains how medication adherence is a dynamic process that changes over time based on the feedback mechanism between health threats (symptom identification) and appraisal of the coping behavior (taking medications) is the Common Sense Model of Illness (Levanthal, Brissette, & Levanthal, 2003). The Common Sense Model emphasizes the influence of social and environmental contexts in behavioral decisions and changes. Thus, based on the Common Sense Model, we hypothesize that as the social and environmental context of older adults change over time (such as retirement or diagnosis of a new chronic condition), their experiences with and perceptions of their diseases and medications may also change. Since these experiences and perceptions may frame, confirm or disconfirm their current beliefs in medicines, we postulate that beliefs in medicines may change over time. Thus, if these beliefs in medicines change over time, then their adherence to medicines could change.

Few longitudinal studies have examined medication adherence and beliefs in medicines over time in specific illnesses such as HIV, depression, rheumatoid arthritis and diabetes (Aikens & Klinkman, 2012; Cooper et al., 2011; de Thurah, Norgaard, Harder, & Stengaard-Pedersen, 2010; Gonzalez et al., 2007; Horne, Cooper, Gellaitry, Date, & Fisher, 2007). While some of the studies measured adherence over time (both baseline and end of the study), they measured beliefs in medicines only at the baseline (Gonzalez et al., 2007; Horne et al., 2007). One study measured both adherence and beliefs in medicines at baseline and at the end of the study, but did not report how beliefs changed over time (de Thurah et al., 2010). Cooper et al, in a study that measured beliefs in antiretroviral therapy (ART) and adherence to two different ART drugs at baseline and at 48 weeks reported a decrease in medication adherence, a decrease in concern beliefs and no change in necessity beliefs in antiretroviral therapy between 4 weeks and 48 weeks (Cooper et al., 2011). Over a six-month study period, Schulz demonstrated that there were significant changes in beliefs in medications among older adults and these changes predicted the change in medication non-adherence; but adherence did not change over time (Schüz et al., 2011). Finally, Porteous et al examined how general beliefs in medications change over a four year period and concluded that they remain stable over time in adults (Porteous, Francis, Bond, & Hannaford, 2010). However, medication adherence was not measured in that study.

The research question for this exploratory study is “how does medication adherence among older adults change over time based on their change in beliefs in medicines?” Understanding these changes is important for health professionals as every health behavior intervention has a temporal component and it is important to know how these changes happen over time. The current study aims to: 1) quantify how medication adherence and specific beliefs in medicine change over time in older adults, 2) examine whether there is a significant difference in patient specific beliefs in medicines and adherence behavior across various age groups and other demographics, and 3) identify the significant predictors of medication adherence over time. The current study builds on our previous study which examined how concern beliefs in medications change over time and what factors affected the change in concern beliefs (Shiyanbola, Farris, & Chrischilles, 2013).

## 2. Method

### 2.1 Design and Subjects

A two-year longitudinal study was conducted using an internet based survey administered by Harris Interactive® (HI). HI maintains a confidential panel of individuals who have opted to be invited to participate in online surveys. Since HI’s panel consists of individuals who have volunteered to take part in surveys, it is a convenience sample. The baseline survey was conducted in October 2005 and the follow-up survey was conducted in October 2007 for the same panel of individuals. The sample consisted of older US adults aged 65 and above, taking at least one prescription medication, and who were enrolled in HI’s online survey panel. Data were weighted to be representative of the total U.S. adult population aged 65 and over on the basis of age, race/ethnicity, education, region, income and propensity to be online. HI data has been used by researchers in the past for healthcare research, including studies on medication adherence (Gadkari & McHorney, 2012; Piette, Beard, Rosland, & McHorney, 2011).

### 2.2 Data Collection

Medication adherence was measured using the validated four-item Morisky scale (Morisky, Green, & Levine, 1986). The items were scored dichotomously (yes(1)/no(0)) and the scores ranged from 0 to 4; lower scores indicate better medication adherence (Morisky et al., 1986). Individual’s beliefs in medicines were measured using the Beliefs about Medicines Questionnaire (BMQ) (Horne, Weinman, & Hankins, 1999). The BMQ scale asks about individual’s opinions about the medicines prescribed to them and comprises of two subscales - specific necessity beliefs in medicines such as “my health at present depends on my medicines” and specific concern beliefs in medicines such as “having to take medicines worries me”. Each subscale has five items on a scale from 1 to 5; the scores range from 5 to 25, higher scores indicates stronger beliefs. A necessity-concern differential was calculated as the difference between individual necessity beliefs and concern beliefs in medicines. When asking questions about medication adherence and beliefs in medicines, the participants were asked to answer the questions regarding their prescription medicines.

The study also collected information on other demographics such as age, gender, and education. For age, the respondents were also classified into various age groups such as 65 to 70, 71 to 75, 76 to 80, and above 80 years old. Data were also collected on self-reported health (ranged from 1 to 5, a higher number indicates better health), and the number of medicines taken on a daily basis. These variables were selected because past studies have shown that they influence medication adherence (Barat, Andreasen, & Damsgaard, 2001; Bosworth et al., 2011; de Thurah et al., 2010; Kennedy, Tuleu, & Mackay, 2008; Murray et al., 2004; Wheeler, Roberts, & Neiheisel, 2014).

### 2.3 Analyses

Descriptive analyses were used to measure medication adherence and beliefs in medicines at baseline and at the end of the study after two years. In addition, Pearson bivariate correlation and t-tests (both independent and paired) were used to determine if there was a significant relationship or correlation between adherence and beliefs in medicines at various time points. Paired t-test was also used to determine if there was a significant change in beliefs in medicines and medication adherence over time in each age group. In addition, one-way ANOVA was used to determine if there was a significant difference in the beliefs in medicines and medication adherence across different age groups.

A linear regression model was used to identify the significant predictors of change in adherence over time. The change in adherence over two years (follow-up adherence subtracted from the baseline adherence) was the dependent variable. The adherence values ranged from 0 to 4 and perfect adherence was defined as a value of 0. The model predicted change in medication adherence using baseline adherence, baseline necessity beliefs in medicines, baseline concern beliefs in medicines, change in necessity beliefs over time, change in concern beliefs over time, change in self-reported health over time, and change in the number of medicines taken regularly over time. These variables were selected since the research question was to determine how change in beliefs in medicines affect medication adherence. In addition, we used change in health and change in the number of medicines as independent variables since these variables can change over time for adults over 65. The model controlled for demographic variables such as age, gender, and education. Analyses were performed using SPSS version 16 (SPSS Inc., Chicago, Illinois, U.S.A).

## 3. Results

Of the 1220 subjects who responded to the baseline survey in 2005 and 1024 subjects who responded to the follow-up survey in 2007, four hundred and thirty six subjects responded to both the baseline and follow-up surveys. Of these 436 respondents, the mean age was 72.54 (SD: 5.49, Range: 65–87) for baseline survey and 74.53 (SD: 5.49, Range: 67–89) for the follow-up survey. 29.5% of the respondents were aged between 65 and 70, 30.9% were between 71 and 75, 22.7% were between 76 and 80, and 16.9% were over 80 years of age. Table 1 has the demographics of the respondents. For the inferential analysis, complete data was available only for 329 subjects. Participant responses with missing data for the required dependent and independent variables were omitted.

**Table 1:** Demographics of respondents in comparison with the baseline and follow-up surveys

Characteristic	2005 respondents	2007 respondents	Respondents to both surveys
	n = 1220	n = 1024	n = 436
Age	72.59 ± 5.7	72.4 ± 5.7	
Women	54.2%	57.8%	55%
Education			
High school or less	18%	19%	16%
Some college	39%	42%	38%
4-year degree and beyond	43%	39%	40%
Non-Hispanic White	91%	94%	92%
Household income < $50,000	66%	51%	56%

The first aim of the study was to examine how medication adherence and specific beliefs in medications change over time in older adults across all the respondents. The baseline medication adherence score was 0.58 and follow-up medication score was 0.59. Both at baseline and at follow-up, approximately 60% of the respondents had a perfect adherence score of 0 and 37% had a medium adherence score between 1 and 2. There was no statistically significant change in adherence (0.58 ± 0.84 vs. 0.59 ± 0.84; p > 0.05), necessity beliefs (17.13 ± 4.31 vs. 17.10 ± 4.29; p > 0.05), concern beliefs (11.70 ± 3.73 vs. 11.68 ± 3.77; p > 0.05), or necessity-concern differential (5.42 ± 5.29 vs. 5.42 ± 5.37; p > 0.05) over the two years. Additionally, for all respondents, there was a significantly strong correlation between baseline and follow-up necessity beliefs (r= 0.656, p < 0.05) and a moderately strong but significant correlation between baseline and follow-up concern beliefs (r = 0.534, p < 0.05). The correlation between baseline and follow-up adherence (r = 0.456, p < 0.05) was also significant, though moderate. When the subset of respondents with less than perfect adherence (adherence score > 0) was analyzed (n = 141), there was a statistically significant improvement in the adherence score (1.45 ± 0.70 vs. 0.99 ± 0.97; p < 0.05). However, there was no difference in either necessity or concern beliefs in medicines. The self-reported health of these individuals also statistically significantly decreased from 3.09 to 2.99 (p < 0.05) and the number of medicines taken on a regular basis increased from 4.56 to 5.09 (p < 0.05).

The second aim of the study was to determine if there is a significant change in the beliefs in medicines and adherence behavior of older adults by age and other demographics. At baseline and over time, there was no statistically significant difference in the beliefs in medicines or adherence behavior by age, gender, and education (data not shown). However, when the respondents were separated as adherent and non-adherent individuals, non-adherent individuals had significantly higher concern beliefs in medications than necessity beliefs at both the baseline (12.84 vs. 10.96; p < 0.0001) and follow-up survey (12.79 vs. 11.05; p < 0.0001).

The third aim of the study was to determine the significant predictors of medication adherence over time (Table 2). The change in adherence over time (baseline adherence – follow-up adherence) ranged from -3 to +3 and the mean was -0.03 ± 0.88. A positive number in the change in adherence over time indicates improved adherence and a negative change in adherence over time indicates worsened adherence over time. While there was no change in adherence over time in 58.8% of the respondents, 21.7% had their adherence decreased over time, and 19.5% had their adherence improved over time. The multiple linear regression resulted in a significant model with an R square value of 32.4%. All the regression diagnostic tests were satisfactory (Table 2 legends). The significant predictors of change in adherence over time were baseline adherence, baseline concern beliefs in medicines, change in health over time (self-reported health decreased over time), and change in the number of medicines over time (increased over time). When the model was analyzed among patients with lower adherence, the same variables predicted the change in adherence over time. As the baseline adherence increased, change in adherence over time also improved. Similarly, as the health decreased over time and the number of medicines increased over time, the adherence improved over time.

**Table 2:** Multiple linear regression analysis to predict change in adherence over time

For all respondents Dependent variable = Change in adherence over time n = 329 (R square = 32.4%)		

Predictors	Co-efficient	P value
Baseline adherence	0.589 (0.466 – 0.650)	0.000
Baseline necessity beliefs in medicines	-0.015 (-0.022 – 0.016)	0.760
Baseline concern beliefs in medicines	-0.149 (-0.056 – -0.009)	0.007
Change in necessity beliefs over time	0.059 (-0.009 – 0.037)	0.233
Change in concern beliefs over time	-0.083 (-0.043 – 0.005)	0.117
Change in health over time	0.110 (0.026 – 0.279)	0.018
Change in the number of medicines over time	0.121(0.013 – 0.106)	0.012

^a^The model controlled for age, gender, and education.

An analysis of standard residuals was carried out, which showed that the data contained no outliers (Std. Residual Min = -2.853, Std. Residual Max = 2.334).

Tests to see if the data met the assumption of collinearity indicated that multicollinearity was not a concern. All the VIF values were lesser than 10, and ranged from 1.035 to 1.467.

The data met the assumption of independent errors (Durbin-Watson value = 2.042).

The histogram of standardized residuals indicated that the data contained approximately normally distributed errors, as did the normal P-P plot of standardized residuals, which showed points that were not completely on the line, but close.

**Table:** 

For respondents with baseline adherence score greater than 0 Dependent variable = Change in adherence over time, n = 130 (R square = 25.7%)		

Predictors	Co-efficient	P value
Baseline adherence	0.413 (0.332 – 0.733)	0.000
Baseline necessity beliefs in medicines	-0.102 (-0.067 – 0.016)	0.232
Baseline concern beliefs in medicines	-0.235 (-0.112 – -0.016)	0.009
Change in necessity beliefs over time	-0.003 (-0.046 – 0.044)	0.973
Change in concern beliefs over time	-0.071 (-0.069 – 0.029)	0.420
Change in health over time	0.177 (0.028 – 0.581)	0.031
Change in the number of medicines over time	0.243 (0.043 – 0.220)	0.004

^a^The model controlled for age, gender, and education.

An analysis of standard residuals was carried out, which showed that the data contained no outliers (Std. Residual Min = -2.158, Std. Residual Max = 2.152).

Tests to see if the data met the assumption of collinearity indicated that multicollinearity was not a concern. All the VIF values were lesser than 10, and ranged from 0.740 to 0.939.

The data met the assumption of independent errors (Durbin-Watson value = 2.015).

The histogram of standardized residuals indicated that the data contained approximately normally distributed errors, as did the normal P-P plot of standardized residuals, which showed points that were not completely on the line, but close.

## 4. Discussion

The aim of this exploratory study was to understand how medication adherence and beliefs in medicines change over time in older adults and what factors predict these changes. The first major conclusion from this study is that both adherence and beliefs in medicines did not change significantly over time in older adults. The authors hypothesized a change in beliefs in medicines would result in a subsequent change in medication non-adherence, based on the mechanism that life changing events would affect beliefs. However, the results from the study did not support the hypothesis. However, for those individuals with initial lower adherence, the adherence improved over time, which was statistically significant. Though there was no change in beliefs in medicines over time for these individuals, the change in adherence might be due to the deteriorating self-reported health and the increase in the number of medicines taken on a daily basis.

The results from this study about older adults in general are similar to the Porteous et al., 2010 study that reported stability in general beliefs in medicines over a period of four years after controlling for the health status of adults (Porteous et al., 2010). Schüz et al measured beliefs in medicines and adherence in older adults with multiple illnesses and reported significant change in beliefs in medicines over a six month period. They also examined how these changes in beliefs predicted non-adherence; but did not report a change in medication adherence (Schüz et al., 2011). Aikens et al examined a change in patient beliefs about their antidepressant drugs over 14 weeks and also reported a change in beliefs, but not adherence (Aikens & Klinkman, 2012). However, when older adults with low adherence were examined, the adherence behavior changed over time.

There can be several reasons for the findings in the current study. First, the baseline medication adherence was high in the current study and there was insufficient variation in medication adherence making it difficult to detect relationships. This may be the reason why a change in adherence behavior was observed only in individuals with low adherence. In addition, we examined participant’s beliefs in medicines in general, and not one specific medicine or disease. Another important factor to be considered for further studies is the clinical significance of these changes. Being mindful of the fact that beliefs in medicines are important in affecting medication adherence, research must begin to focus on the amount of change in beliefs in medicines that is needed to create a clinically significant change in adherence behavior.

The second major conclusion from the study is that it is important to address the concern beliefs of older adults at the beginning of therapy to secure acceptable medication adherence. The regression analysis of this study demonstrated that baseline concern beliefs in medicines and baseline adherence play a significant role in determining the change in adherence over time in older adults. Additionally, individuals with lower levels of adherence had significantly higher concern beliefs in medicines than necessity beliefs at both the baseline and follow-up survey. Furthermore, the correlation between concern beliefs in medicines and non-adherence was significant at both baseline and follow-up surveys. An earlier literature review that examined evidence-based interventions that can be recommended to improve medication adherence, suggested that the initiation of such programs should be at the time of therapy initiation when patients are forming attitudes and beliefs about medications and adherence (Petrilla, Benner, Battleman, Tierce, & Hazard, 2005). In addition, our study results are in agreement with the expert consensus on the need to identify suboptimal non-adherence in the earliest stages so that it can be addressed (van Dulmen, 2008).

Though the study brings forth an important implication in adherence interventions for older adults, it is not without limitations. The study was conducted using a convenience sample from an online panel of individuals and is not generalizable. In spite of all the measures adopted by Harris Interactive to reduce bias, selection bias is still a possibility. Also, the study did not measure the medication adherence of an individual with a specific chronic disease. This is significant since recent research suggests that medication adherence varies by medicines (Krigsman, Nilsson, & Ring, 2007; McHorney & Gadkari, 2010). Though the study assumed that changes happen in the life of older patients (such as retirement, or diagnosis of a new condition), it was not directly measured in the study. This study only examined how change in beliefs in medicines affects adherence behavior over time. Other factors such as a change in illness perceptions or self-efficacy might affect a change in adherence behavior. However, these factors were not measured in this study. The study participants’ medication adherence was measured using a dichotomous response of Yes/No versus a continuous response scale. In contrast, the beliefs in medicines were measured on a continuous scale. Hence, the statistical relationship between beliefs and adherence might not be observable.

The current adherence research has established the factors that affect medication adherence. However, future research should focus on identifying the factors that influence change in adherence behavior over time since understanding the temporal component of medication adherence is important while designing interventions to improve medication adherence. Based on the Common Sense Model of Illness, we do not know if social relationships, cultural norms or other types of information received might affect adherence behavior over time. Though the study hypothesis was based on the Common Sense Model, the study did not directly measure the illness perceptions of older adults. The study measured patient beliefs in medicines which is a key part of the Extended Common Sense Model for medication adherence (Levanthal et al., 2003). A next step would be to determine if the illness perceptions of older adults change over time and whether a change in their illness perceptions has an impact on their beliefs in medicines and medication adherence.

### 4.1 Practice Implications

The major practice implication from these study findings is the recommendation to use interventions and counseling that will address the concern beliefs in medicines of older adults at the beginning of therapy so that appropriate beliefs that will support medication adherence are developed early. Although this recommendation may seem to oversimplify the reasons for non-adherence in older adults, based on the study findings, it is known that an increase in the baseline concern beliefs in medicines was directly related to the decrease of medication adherence over time. This observation does not suggest that current interventions provided to older adults in improving adherence, such as memory aids or improving the knowledge and skills of medication taking, are not valid. Instead, it reinforces the need to provide theory-based interventions that addresses all aspects of non-adherence including beliefs in medicines and personal experiences early on in medication therapy as suggested in the literature (Petrilla et al., 2005; Ruppar, Conn, & Russell, 2008; van Dulmen, 2008).

## 5. Conclusion

With an increasing number of older adults in the health care system, it is important to understand how their medication adherence behavior and beliefs in medicines change over time. This study demonstrated that for all older adults, there was no significant change in adherence over time. However, for the older adults with initial low adherence, adherence improved over time. Baseline adherence and baseline concern beliefs in medicines were significant predictors of this change. Thus, it is important to alleviate older adults’ concern beliefs at the beginning of therapy for better adherence.
